# Creating strong predictive models in oncology

**DOI:** 10.1016/j.patter.2026.101492

**Published:** 2026-02-13

**Authors:** Michael F. Gensheimer

**Affiliations:** 1 Stanford University School of Medicine, Palo Alto, CA 94304, USA

## Abstract

Many oncology predictive models fail to improve care. Issues include risks of bias, underpowered radiomics studies, and limited clinical impact. A path forward involves an emphasis on clinically actionable questions, rigor, and generalizability.

## Main text

As technology marches forward, one can start to see a vision of precision oncology care from the near future. The patient is diagnosed at an early stage using a combination of blood DNA sequencing and advanced high-resolution imaging. Their initial treatment is chosen with the assistance of a sophisticated machine learning model that uses features from their MRI scan that would be imperceptible to humans, along with the specifics of the tumor’s DNA mutations, to simulate what would happen if different treatments were given in this exact situation. They are given a customized amount of radiation, with the treatment ending as soon as real-time biomarkers indicate an acceptable cancer control rate, enabling side effects to be minimized. Their follow-up care, including automated questionnaires and education materials, is tailored to their personalized risk of developing a recurrence or various side effects.

I believe that many of these things will become a reality and will make a difference for patients. Much of my research as a radiation oncologist has focused on predicting patient outcomes using imaging and other clinical data, such as the text of clinicians’ notes, which will be needed in order to customize care. However, I am concerned that much of the research effort in this space is misplaced and may not result in improved care for our patients.

### Two requirements

With the advent of large electronic health record datasets, digitized imaging and pathology, and mutation panels like FoundationOne (https://www.foundationmedicine.com/), it is easier than ever to find significant associations between clinical features and outcomes, create prediction models, and publish papers based on the findings. But there is reason for caution. After all, everything correlates with everything else, especially in living organisms where there are intricate interactions between genes and environment. For a prediction model to be useful, two things must be true: it should reliably improve predictive performance compared with alternatives, and based on the result, we should be able to change something that makes a positive impact on the patient or the health system. To ensure high and reliable performance, authors should follow steps like preregistering their analysis plan, using a large enough sample, reporting in detail on the patient population and analysis methods, and, ideally, making their code and data available. To ensure clinical impact, honest conversations with forward-thinking clinicians can go a long way.

A recent dramatic example outside of cancer where these goals were not met was the flood of papers during the COVID-19 pandemic trying to predict COVID-19 infection using deep learning models trained on chest radiographs (a PubMed search reveals over 1,000 papers on this topic). A review of these papers found high risk of bias from small sample size and lack of calibration metrics. Most of the papers also had serious methodological issues; for instance, a majority did not describe basic details about the patient population, such as how patients were selected for inclusion, which makes it impossible to know whether the performance metrics would apply to a new setting.^1^ Another major issue is that COVID-19 testing from nasal swab samples is quite accurate and gets rapid results, so the clinical need for COVID-19 diagnosis from chest radiographs is low. As far as I am aware, none of these models ended up being deployed clinically.

### Radiomics

Many predictive models in cancer suffer from similar issues. Similarly to trying to diagnose COVID-19 from chest radiographs, there has been great interest in using imaging like computed tomography (CT) scans to predict molecular features or outcomes of cancers, known as radiomics. The hypothesis is that there are imaging features of tumors that are not perceptible to humans but indicate biological differences and predict cancer outcomes or response to treatment. There have been almost 8,000 papers on radiomics in oncology, but remarkably few clinical trials using radiomics models in actual patients have been performed, and few models are in clinical use.^2^

Because I have been involved in a number of radiomics papers, my Google Scholar new articles feed features many radiomics papers each week. I picked one of this week’s papers at random, and it highlights well many of the common issues with these studies.^3^ I cite it only because it is representative: it is a fairly typical radiomics study and not unusually flawed, and the limitations I discuss are widespread in the literature. The authors wished to predict which lung metastases would have a complete response (disappearance of visible tumor on CT) after high-dose external beam radiation treatment. They used radiomics on the pretreatment CTs and emphasized “explainable artificial intelligence” so the end user can see which features influenced each tumor’s predicted outcome. They found an area under the receiver operating characteristic curve (AUC) of 0.86 on the test set, which sounds very promising. Unfortunately, there are some major issues. Patient and tumor characteristics, such as cancer types and tumor sizes, were never mentioned, but these are important to understanding the expected level of treatment response. Eighty tumors were included, with an 80%/20% train/test set split; there was a 61% complete response rate in the overall dataset. This means that there were around 26 events (lack of response) in the training set used for fitting the model and 6 events in the test set used for assessing performance. There were 107 radiomics features and at least five clinical features used for model training, for a features-to-events ratio of 4:1. The tiny number of events is woefully inadequate to reliably estimate the effect of all of these variables on response—there is just not going to be enough signal.^4^ It seems highly likely that in a separate bigger sample, largely different features would be selected as significant. Another way of emphasizing the small amount of outcome information available is that in the entire dataset of 80 tumors, there were 80 bits of tumor outcome information (the equivalent of 12 ASCII characters). This is not enough to extract any sort of structure from the image data.

### Clinical impact

This can be contrasted with large language models, which are trained on trillions of bits of next-token outcome data, or with medical image segmentation models that get to use the millions of voxels in each CT image as outcome data and so can be trained on surprisingly few images (the famous U-Net paper used only 30 images for training).^5^ Since radiomics papers typically are not preregistered, do not share analysis code or data, and often report high AUC on small samples, many readers will be concerned that their results will not replicate in a larger independent sample.

Clinical impact is the other critical requirement for a predictive model to be useful. Most of these models are designed to be used to make better treatment decisions, to try to improve cancer control or reduce toxicity. The stark reality is that even with a high-performance model or biomarker, it is not easy to make a clinical impact. This is because human biology is complex and still somewhat mysterious, and it is very hard to predict the benefit of a treatment change without doing a clinical trial in hundreds of patients over many years. An example is human papillomavirus (HPV) infection in oropharynx cancer, a cancer type I treat often. Patients whose cancers are HPV positive have a much better prognosis than those with HPV negative tumors, so HPV status is an important biomarker. Since there is a high cure rate for these HPV positive tumors, we have been trying for over 15 years to find ways to de-escalate the treatment compared with HPV negative treatment in order to reduce side effects. Unfortunately, there have been several high-profile trials that showed inferior cancer control with de-escalation, including RTOG 1016 and NRG-HN005, and the standard of care remains 70 Gy of radiation over 7 weeks with cisplatin chemotherapy, the same treatment that we have been using for decades and the same treatment as for HPV negative tumors.

Another factor that reduces clinical impact is that many predictive modeling studies in oncology aim at targets that are not very clinically relevant. For instance, there are many papers trying to predict whether a non-small cell lung cancer has an EGFR mutation based on radiomics on CT imaging. But there are big problems with this. The performance level of these radiomics models is not high enough to make treatment decisions, and from the body of literature on radiomics, it is hard to see a path toward adequate performance for this task even in the future. There is a very strong alternative approach, which is next-generation sequencing on a biopsy specimen that indicates not just whether there is an EGFR mutation but the specific subtype. And the treatment landscape is moving quickly: there are now treatment approaches that depend on specific EGFR mutation types like exon 20 insertions, which the radiomics models are not able to predict.

### High-signal biomarkers

One path to clinical relevance is to develop predictive models with exceptionally high performance so that we can confidently change treatment based on the results. A recent example is circulating tumor DNA (ctDNA) analysis after curative intent treatment of lung, colon, and other cancers. The seminal papers showed that detectable post-treatment ctDNA has an eye-popping ability to predict later cancer recurrence, with hazard ratios in the 10–50 range. Now, 5 to 10 years after those initial papers, clinical trials using ctDNA to choose adjuvant treatment are starting to be reported, with some positive and exciting results.^6^

An easier option is to keep the model simple and restrict the predictor variables to ones that have known biological validity or are clinically established. Mirels’ score is a good example of this. This score predicts the risk of a bone metastasis causing a fracture, and it uses only four features (lesion size, site, appearance such as bone erosion, and pain). It has modest predictive ability, but it is easy to calculate in the clinic using an online calculator, and the predictors have biological plausibility and match clinicians’ experience. So it is commonly used in the clinic and in research.

### A path forward

In the common scenario where a researcher wants to develop a more complex predictive model and it’s not expected to have blockbuster predictive performance like ctDNA, what is the path to a useful, practice-changing model? Ideally, the work starts with in-depth conversations with an engaged, expert clinician. The team should model what would happen if the predictive tool were used in practice, with reasonable assumptions about performance and the effect of model-informed treatment selection on cancer outcomes. AI coding tools make it much easier to do this, including simulation-based sample size calculations. Recent papers offer good guidance on performance metrics and validation.^7^ This up-front work will help decide whether single-institution data is sufficient or whether a larger multi-institutional dataset is needed. Once the project has started in earnest, the researchers should preregister the analysis and then report the methods in enough detail that someone could reproduce it. They should follow the checklist in a statement like TRIPOD+AI to ensure rigor and should release their analysis code and, ideally, anonymized data as well.^8^ These things will not guarantee that the model will be impactful, but they greatly increase the chances.

If we focus on generalizable performance and clinical usefulness—the core features of a strong predictive model—we can accelerate work that truly helps patients and spend less time on models that were never likely to influence practice.

## Declaration of interests

M.F.G. reports stock ownership in Amgen.
